# Perspective on utilization of *Bacillus* species as plant probiotics for different crops in adverse conditions

**DOI:** 10.3934/microbiol.2024011

**Published:** 2024-03-13

**Authors:** Shubhra Singh, Douglas J. H. Shyu

**Affiliations:** 1 Department of Tropical Agriculture and International Cooperation, National Pingtung University of Science and Technology, Pingtung 912301, Taiwan; 2 Department of Biological Science and Technology, National Pingtung University of Science and Technology, Pingtung 912301, Taiwan

**Keywords:** *Bacillus* species, crops, endophytes, plant growth-promoting bacteria, plant probiotics, sustainable agriculture

## Abstract

Plant probiotic bacteria are a versatile group of bacteria isolated from different environmental sources to improve plant productivity and immunity. The potential of plant probiotic-based formulations is successfully seen as growth enhancement in economically important plants. For instance, endophytic *Bacillus* species acted as plant growth-promoting bacteria, influenced crops such as cowpea and lady's finger, and increased phytochemicals in crops such as high antioxidant content in tomato fruits. The present review aims to summarize the studies of *Bacillus* species retaining probiotic properties and compare them with the conventional fertilizers on the market. Plant probiotics aim to take over the world since it is the time to rejuvenate and restore the soil and achieve sustainable development goals for the future. Comprehensive coverage of all the *Bacillus* species used to maintain plant health, promote plant growth, and fight against pathogens is crucial for establishing sustainable agriculture to face global change. Additionally, it will give the latest insight into this multifunctional agent with a detailed biocontrol mechanism and explore the antagonistic effects of *Bacillus* species in different crops.

## 1. Introduction

Chemical fertilizers are widely used in current farming systems worldwide to meet the growing demand for food despite their high cost and severe harmful consequences for the environment and human health. Probiotics are living bacteria that provide the host with health benefits when given in sufficient doses. The term was initially used concerning the interactions of microorganisms with either animal or human hosts [1]. However, due to their wide availability and emerging technologies, researchers are interested in learning about the interaction of these beneficial microorganisms with plant growth and development. It is interesting to note that several studies show that probiotics could enhance crop production and improve foods' nutritional levels including antioxidant properties [2]. Therefore, these bacterial species are considered to belong to plant growth-promoting rhizobacteria (PGPR). They are responsible for improving the health of plants and have been known as plant probiotics in recent years. Plant probiotics work through direct or indirect mechanisms at physiological levels. Plant-associated microorganisms are reported to enhance the solubilization of fixed phosphorus to available forms for improving plant yields. This term covers probiotic bacteria in the phylum, such as *Actinobacteria*, *Bacteroidetes*, *Firmicutes*, and *Proteobacteria* [3]. This review focuses on the growth-promoting potential of *Bacillus* species involved in different crops to use these beneficial species as probiotics under biotic and abiotic stresses.

## 2. Plant growth-promoting *Bacillus* species as plant probiotics

Gram-positive *Bacillus* species are common in nature and can be found in practically all environmental niches. Additionally, these species have been utilized to produce industrial, agricultural, and medical goods [4]. Chemical fertilizers and pesticides can be replaced with antagonistic bacterial strains as biofertilizers. They can offer fresh perspectives on improving plant growth and productivity in case of diseases or pest infestation. For example, a rhizospheric *Bacillus* strain native to Kerala, India, shows an antifungal effect towards *Pythium myriotylum* in addition to the chemical compound responsible for antifungal activity [5]. A rhizospheric *B. subtilis* strain isolated from Himalayan regions was found to produce the antifungal antibiotic iturin A, a lipopeptide which inhibited growth of the phytopathogenic fungi *Fusarium oxysporum* and *Rhizoctonia solani* [6],[7]. This strain could protect tomato plants from fungal diseases and increase fruit yield. Evidently, certain *B. subtilis* strains isolated from the rhizosphere of desert plants produced volatile organic compounds such as 2,3-butanediol, which induced systemic resistance in *Arabidopsis* plants [7]. This triggered immunity against the bacterial leaf pathogen *Pseudomonas syringae*, reducing disease incidence. A strain of *B. subtilis* from potato rhizosphere showed ability to protect potatoes from common devastating scab disease caused by *Streptomyces scabies* by the production of lipopeptides surfactin and iturin A, which inhibited germination of the pathogen's spores [8]. Another endophytic strain of *B. subtilis* ME9 isolate from cassava showed a broad-spectrum antibacterial compound that inhibited bacterial leaf streak caused by *Xanthomonas phaseoli* pv. *Manihotis*, reducing disease severity and improving cassava yields [9]. Similarly, a plant growth-promoting *B. subtilis* strain obtained from semi-arid regions in Africa demonstrated salt stress tolerance and could mitigate the effects of salinity on sorghum growth through production of osmolytes and modulation of stress-responsive genes [10].

As mentioned, the mechanism behind the growth-promoting effects of *B. subtilis* is associated with probiotic properties such as biofilm formation. The biofilm formation of *B. subtilis* was regulated by specific genes in plant polysaccharides [11]. A biofilm-forming *B. subtilis* strain from Brazil was capable of solubilizing insoluble phosphorus in the soil and releasing it to promote increased growth and productivity in corn, wheat, and other plants in phosphorus-deficient soils [12]. These biofilms provide numerous beneficial effects that stimulate the growth and health of plants. One key mechanism is nitrogen fixation, whereby *B. subtilis* converts inert atmospheric nitrogen into a usable form of nitrogen that plants can incorporate for growth. The biofilms also solubilize insoluble phosphorus in the soil through organic acid production, making this important macronutrient plant available. Another growth-promoting effect is the biosynthesis of antimicrobial compounds by the biofilms that protect plants against infection by fungal, bacterial, and viral pathogens. Beyond direct nutrient contributions and pathogen defense, *B. subtilis* and *B. amyloliquefaciens* biofilms also induce systemic resistance in plants, priming their innate immunity to fight off diseases more effectively [13],[14]. Therefore, *Bacillus*-based biofertilizers can be directly applied to the surface of the soil for the enhancement of the control of microbial growth of disease-causing pathogens. Moreover, the effects of the induction of a pest defense system and the increased availability of plant nutrients in rhizospheres were observed [15].

## 3. Biocontrol mechanism of *Bacillus* species in stressful conditions

**Figure 1.**
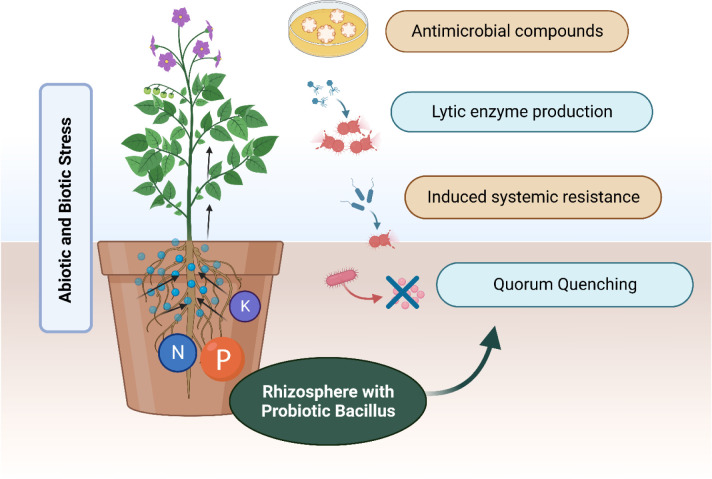
Effect of *Bacillus* on plant protection in biotic and abiotic stress. Illustration depicting the multifaceted impact of *Bacillus*-based probiotic mixture on rhizosphere can help plant to restore nitrogen (N), phosphorus (P) and potassium (K) and overcome biotic and abiotic stresses by the production of antimicrobial compounds, lytic enzymes, quorum sensing molecules or imparting induced systemic resistance (Created with Biorender.com).

*Bacillus* species can promote growth and development in stressed conditions through complex mechanisms, which can be grouped into various modes of action, as mentioned in Figure 1 [15],[16]. *Bacillus* as plant probiotics have developed an arsenal of strategies to aid plants in withstanding stressful conditions that would otherwise inhibit growth and cause damage. As mentioned above, under stress such as drought, certain *Bacillus* strains provide drought resilience by synthesizing the stress hormone abscisic acid, which triggers adaptive water-conserving responses in plants like stomatal closure to reduce water loss through transpiration. They also form exopolysaccharide biofilms that help retain soil moisture and prevent desiccation [17]. For salinity tolerance, *Bacillus* equip plants through production of the enzyme, 1-aminocyclopropane-1-carboxylate (ACC) deaminase, which cleaves the ethylene precursor ACC to lower deleterious ethylene levels induced by high salts. It also facilitates osmolyte accumulation in plant cells to maintain turgor pressure and physiological functions when stressed by hyperosmotic conditions [18],[19]. In chilling temperatures, *Bacillus* sp. JC03 secrete volatile organic compounds such as auxin and strigolactone that systemically induce biomass accumulation in *Arabidopsis*, promoting plant growth. They also modulate the formation of protective ice nucleating proteins to prevent intracellular freezing and frost injury [20]. Finally, in high heat conditions, *Bacillus* strains HT1 to HT4 isolated from Saudi Arabia elicit the plant's own antioxidant systems to scavenge dangerous reactive oxygen species produced as a result of heat stress. They also synthesize beneficial heat shock proteins that confer protein stability and prevent misfolding when plants experience spikes in temperature [21]. Through these diverse mechanisms related to plant hormones, osmolytes, protective proteins and other compounds, *Bacillus* species provide plants the biological means to withstand adverse environmental conditions and continue thriving.

Therefore, as shown in the subsequent figure, biocontrol mechanisms of *Bacillus* species include a myriad of functions:

- Biological nitrogen fixation
- Stress-mediated enzymes, such as 1-aminocyclopropane-1-carboxylate (ACC) deaminase, and extracellular enzymes for hydrolyzing the cell wall
- Plant growth regulators, such as abscisic acid (ABA), cytokinins (CKs), gibberellic acid (GA), and indole-3-acetic acid (IAA)
- Siderophore production for the chelation of available iron in the rhizosphere
- Micronutrient and macronutrient biosolubilization and biomineralization, especially converting insoluble phosphate precipitates to soluble forms
- Biocontrol of plant pathogens by other mechanisms, such as antibiotics production, induced systemic resistance (ISR), quorum quenching, and microbial competition for metabolic niches within the rhizosphere
- Agricultural soil structure improvement and contaminated soil bioremediation, such as toxic heavy metal species sequestration and xenobiotic compounds degradation
- Abiotic stresses resistance enhancement [22]

Nitrogen synthesis is essential for plant growth and development since forming amino acids and proteins is considered an important biochemical pathway for synthesizing the macronutrients for plants [23]. Specific organic molecules produced by some microorganisms, such as phytohormones, are involved in plant photomorphogenesis. However, some beneficial bacteria or probiotic species are involved in plant growth enhancement and disease resistance [24],[25]. For example, auxins are responsible for regulating bacterial communication and coordinating other functions of plants directly or indirectly, and some act as signaling molecules for bacterial communication. An auxin-producing *Bacillus* species WM13-24 and a *B. amyloliquefaciens* GB03 which were isolated from the rhizosphere of *H. ammodendron* were developed as biofertilizers. They were found to enhance the growth, fruit yield, and quality of *Capsicum annuum* with improvement in nitrogen content, enzyme activities, and biodiversity of viable bacteria for long-term effects [26].

Similarly, *Bacillus megaterium* with *Azotobacter chroococcum* are found to promote cucumber growth by producing cytokinins. Cytokinins are responsible for cytokinesis, vascular cambium sensitivity, root apical dominance, and vascular differentiation [27],[28]. Under stressed conditions, plants often produce ethylene that can inhibit specific processes that cause premature senescence, such as root elongation or nitrogen fixation in legumes. Some bacteria produce ACC amidase to hydrolyze ACC, the precursor molecule involved in the biosynthesis of ethylene. Therefore, these probiotic species lower ethylene levels and protect the plant from damage [2]. Plant growth and germination of seeds are affected by nutrient availability. Generally, root transporters are responsible for the absorption of soluble phosphorus and nitrogen forms from the soil. However, the rhizosphere is the bioavailable center for P and N [29]. Some effects of the beneficial *Bacillus* species and their bioactive components are shown in Table 1. *Bacillus* species can convert the complex forms or the precipitate forms of essential macronutrients to the available soluble forms for the plants during uptake by plant roots. Some of the reported strains of *Bacillus pumilus* and *B. subtilis* can positively fix available nitrogen, solubilize the phosphate, and produce auxins during in vitro studies [30],[31]. It is reported that *Bacillus* species have *nifH* gene and produce nitrogenase involved in nitrogen fixation to delay senescence and enhance plant growth and yield [30]. In the pursuit of sustainable agriculture, researchers are exploring innovative strategies to improve soil fertility and one promising eco-friendly approach involves the combination of biochar and beneficial microorganisms, such as *B. subtilis* SL-44. This synergistic approach not only addresses soil health but also contributes to increased agricultural productivity [57]. SL-44, also known for its antagonistic properties against fungi, presents an opportunity for the isolation, purification, and identification of antifungal proteins. Studies have elucidated the antifungal protein produced by *B. subtilis* SL-44 has potential application in addressing anti-fungal resistance in apple cultivation [58]. Subsequently, studies have found that the enhancement of chromium contamination can be remediated by SL-44 strain through the addition of humic acid (HA). The study also has suggested dual function of HA on SL-44 species in Cr (VI) reduction, augmentation of Cr (VI) complexation, adsorption, and electronic exchange reduction in the contaminated soil [59].

**Table 1.** Plant growth-promoting characteristics with *Bacillus* species.

Types of species	Growth promoting characteristics	Bioactive metabolites/other contents	Inferences	Reference
*Bacillus* sp. LKE15	Increased shoot length, root length, and plant fresh weight of the oriental melon	Increased synthesis of chlorophyll and individual amino acids along with magnesium, potassium, and phosphorus content	Cell-free *Bacillus* sp. LKE15 on crop plants has excellent potential as an organic method of improving plant growth	[32]
*Bacillus* sp. WG4	Plant growth-promoting properties and antifungal effects against *Pythium myriotylum*	Pyrrolo [1,2-α] pyrazine-1,4-dione, hexahydro-3-(phenylmethyl)	Promising application as an antifungal plant probiotic agent alone or in combination with other agents like *Trichoderma*	[5]
*Bacillus subtilis* BA-142, *Bacillus megaterium*-GC subgroup A. MFD-2	Foliar application increased fruit length and mineral contents of tomato and cucumber fruit	none	Great potential to increase the yield, growth, and mineral contents of tomato and cucumber vegetable species	[33]
*Bacillus subtilis* LDR2	Promoting plant growth through improved colonization of beneficial microbes under drought stress in *Trigonella* plants	High levels of ACC deaminase and reduced ethylene concentrations	Beneficial for legumes cultivated in arid conditions	[34]
*Bacillus insolitus* (strain MAS17), and *Bacillus* sp. (strains MAS617, MAS620 and MAS820)	Increased the dry matter yield of roots (149–527% increase) and shoots (85–281% increase), and the mass of RS (176–790% increase) in pot experiments	Exopolysaccharides (EPS) productions	EPS-producing bacteria could serve as a valuable tool for alleviating salinity stress in salt-sensitive plants	[35]
*Bacillus megaterium* mj1212	Increasing shoot length, root length, and fresh weight in mustard plants	Increased chlorophyll, sucrose, glucose, fructose, and amino acids content	Phosphate biofertilizer to improve the plant growth	[36]
*Bacillus methylotrophicus* KE2	Enhance shoot length, shoot fresh weight, and leaf width of lettuce	Increased gibberellins and indole acetic acid contents along with high nutritional content	A potential candidate for increasing nutritional contents	[37]
*Bacillus mojavensis*	Increase the dry weight of root and shoot, chlorophyll content, and nutrient uptake in salt-stressed wheat plants	Bacteria containing ACC deaminase	*Bacillus mojavensis* could be an effective strain to promote the growth of wheat in saline soils	[38]
*Bacillus subtilis* (HYT-12-1)	37%-IAA production; 37%-phosphate solubilization; 24%-siderophores production; 85%-potential nitrogen fixation; 6%-ACC deaminase activity in tomato seeds	High ACC deaminase activity in gnotobiotic and greenhouse conditions	*B. subtilis* strain HYT-12-1 would have great potential for industrial application as a biofertilizer in the future	[39]
*Bacillus* sp. CaB5	Enhancement effect on seed germination as well as plant growth in cowpea (*Vigna unguiculata*) and lady's finger (*Abelmoschus esculentus*)	None	Potential of CaB5-based formulation for field application to enhance the growth of economically important plants	[40]
*Bacillus vietnamensis*	Plant growth and bud development with antifungal, anti-inflammatory, anticancer, and antibacterial activities in ginger rhizome against *Pythium myriotylum*	2,4-bis (1,1-dimethylethyl) phenol	Biocontrol agent for *Pythium* rot in ginger as an alternative to fungicides	[41]
*Bacillus subtilis* CBR05	Increased total phenol and flavonoid contents of tomato fruits along with in tomato fruits	Stimulated antioxidant activities and levels of carotenoid (β-carotene and lycopene) content	Biofertilizers-based on PGPR may be a viable alternative to improve the nutraceutical quality of greenhouse-produced tomato fruits	[42]
*Bacillus cereus* QJ-1 bio-organic fertilizer (BOF)	Reduces tobacco bacterial wilt disease and improves soil quality	High nitrogen, phosphorus, and organic matter in rhizosphere soils	BOF can improve the ecological stability of soils and mitigate tobacco bacterial wilt disease	[43]
*Bacillus* biofortified organic fertilizer (BOF)	Disease incidence was lowest (10.7%) in BOF treatment when compared to OF (organic fertilizer) treatment (13.1%), along with an increase in the shoot and root length in tomato plants	None	Management of bacterial wilt disease under poly house conditions of Andaman Islands	[44]
*Bacillus cabrialesii* BH5	Control the fungal pathogen *Botrytis cinerea* in tomato rhizosphere	Cyclic lipopeptide of the fengycin family	BH5 and fengycin H are up-and-coming candidates for biological control of *B. cinerea* and the associated gray mold	[45]
*Bacillus pumilus* 104	Inhibited the growth of *Phytophthora nicotianae* and *P. palmivora* in citrus trees	Antifungal properties with antimicrobial compounds	None	[46]
*Bacillus amyloliquefaciens* Ba168	Inhibited growth of blue mold caused by *Penicillium expansum* in apples	Flagellin an antifungal compound	Flagellin is one of the essential antimicrobial substances from Ba168	[47]
*Bacillus amyloliquefaciens* Ba01	Inhibited the growth and sporulation of *Streptomyces scabies* in potatoes	Surfactin, iturin A, and fengycin	*Bacillus* species control potato common scab in nature	[48]
*Bacillus amyloliquefaciens* FZB42	Antibacterial activity against *Xanthomonas oryzae* rice pathogens	Difficidin and bacilysin	Environmentally friendly and versatile in their mode of action	[49]
*Bacillus amyloliquefaciens* Ar10	Antagonistic activity against bacterial soft rot of potato caused by *Pectobacterium carotovorum* II16	Glycolipid-like compounds	Best alternatives for compounds against the soft rot disease of potato	[50]
*Bacillus subtilis* V26	Effective against root canker and black scurf Tuber (*Rhizoctonia solani*) on potato	Chitosanase and proteases	Great potential to be commercialized as a biocontrol agent	[51]

Other mechanisms, such as cell wall modification, metabolic response changes, and gene expression alteration, occur during stressful conditions. Therefore, researchers focus on signaling molecules and expression analysis to downstream the bioactive metabolites and genes in plant-microbe interaction [52]. Quorum sensing involves cell-to-cell signaling mechanisms to regulate the pathogen population and virulence gene expression. It is considered a crucial process that enables host colonization and microbe survival under stressful situations [53]. Some microorganisms, such as *B. cereus* and *B. thuringiensis*, secrete volatile metabolites. These metabolites, such as alkyl sulfides, indoles, and terpenes, were involved in the quorum quenching mechanism, disrupt the signaling of the pathogens in the soil microbiome, and protect the plant from diseases such as soft rot in potatoes, carrots, etc. [54],[55]. Some microorganisms, such as *Bacillus* and *Serratia* synthesize pigments that have the ability to filter radiation to prevent the damage of nucleic acids under high light intensity [27]. *Bacillus* sp. uses a proton transfer system in the cytoplasm to maintain osmotic balance during pH fluctuation conditions. The regulation of cellular metabolite activities can control protein function, increase cellular vitality, and inhibit pathogen growth. Mechanisms such as solubilization, chelation, modifications, and oxidation-reduction reactions were commonly utilized [56].

## 4. Role of antimicrobial bioactive compounds in biotic and abiotic conditions

*Bacillus* species can induce systemic resistance in plants and increase the uptake and translocation of pesticides in the plant cells of the root system to control pest infestations [60]. The bacterial infection starts with the release of crystal proteins to damage the larval midgut epithelium of the insects at the primary site, interacting with chitin and damaging the peritrophic membranes. For instance, *B. thuringiensis* Cry1 protein domain III damages the peritrophic membrane of Asian corn borer [61]. In addition, *B. thuringiensis* synthesized lipopeptides and polyketides in the later stages of infection. The compounds, such as bacillaene, bacillomycin, difficidin, fegycins, iturin, macrolactin, and surfactin, could modify the process of vacuolization, induce the formation of vesicles, cause the lysis of cell membranes, damage the microvilli, and subsequently lead to the death of larvae [51],[62]. In the cellular phospholipid bilayer, protein surfactin is attached to calcium receptors and changes the peptide composition, while iturin forms ion-conducting pores and increases cell membrane permeability [63]. *Bacillus* species suppress the pathogenic microbial populations that infect and cause diseases of plants, such as *Pseudomonas savastanoi*, *Ralstonia solanacearum*, and *Xanthomonas axonopodis* [64],[65]. *Bacillus* species form biofilm around the root surface and secret polyketides to destroy the membranes of pathogens and reduce diseases by changing the morphology of cell walls and then killing the pathogens [11],[35].

## 5. Comparative study of *Bacillus*-based fertilizers with conventional fertilizers and pesticides

The viability of microbial consortia of the biofertilizer depends on the inoculation method applied. It influences the establishment of microbial populations in the rhizosphere along with the structure and function of microbial populations. Microcapsules have been studied as the best alternative to protection and controlled release [66]. The commonly used carrier materials include inorganic materials, such as charcoal, clay, compost, rock phosphate pellets, talc, vermiculite, and zeolite, and organic materials, such as diatomaceous earth, sawdust, rice bran, and wheat bran [16]. Proper formulation is key to a successful and effective biofertilizer. A comparative chart (Table 2) highlights the advantages of *Bacillus*-based biofertilizers over conventional chemical fertilizers:

**Table 2.** Comparison chart highlighting the specific advantages of *Bacillus*-based fertilizers.

**Parameters**	***Bacillus*-Based Biofertilizers**	**Conventional Chemical Fertilizers**
Nutrient Delivery	Provide nutrients through biological nitrogen fixation, P solubilization, phytohormone production	Provide inorganic forms of NPK only
Soil Health	Improve soil structure, organic matter; no accumulation of salts or chemicals	Can degrade soil quality over time, salinization
Environmental Impact	Eco-friendly, no toxic chemical residues	Leaching causes water pollution, eutrophication
Effectiveness	Nutrients available over longer-term; activate soil microbiome	Nutrients easily leach away, may require frequent re-application
Plant Growth	Promote extensive root development, stress tolerance	Primarily provides macronutrients only for growth
Cost	Lower cost per acre over long term	Petroleum derived, energy intensive high cost over time
Safety	No health risks; biopesticides improve food safety	Risk of toxic metal accumulation; pesticide residues
Yield	Increased yields due to well-developed healthy plants	Lower yields over time as soil quality declines
Regulatory Approval	Considered safe; no restrictive regulations	Subject to government health and safety regulations

Natural carriers such as bacterial species must possess the capability of long-term viability; however, the nutrient competition makes it more challenging to adapt to certain micro-habitats. This natural selection among pathogens challenges the probiotic bio-based fertilizers [67]. In addition to the carrier materials, the inoculum density and the response of the plant towards the inoculators are highly influenced by root colonization that varies from bacterial division rate and distribution in the rhizospheres [66]. Another significant issue is acclimatization to the new environment, which leads to a decrease in the microorganism population. Accompanied by biotic and abiotic factors, it affects the structural and functional diversity of microorganism communities. In this manner, a study of the evaluation of probiotic inoculants towards site-specific plant associations could determine the fate and activity in the soil [68].

Organic farming is an integrated farming system where bacterial agents are considered eco-friendly and sustainable for increasing soil fertility and protecting plants against diseases [69]. Plant probiotic *Bacillus* species can promote organic farming and reduce farmers' dependence on chemical fertilizers and promote sustainable farming in modern agriculture. For instance, an alternative crop production technique was applied by the inoculation of both *B. subtilis* FZB24 and GB03 in corn (*Zea mays* L.) seedlings. They reduced the use of neonicotinoid insecticide thiamethoxam. Similar studies have been observed with the evaluation of using thiamethoxam in tomato crops [70]. The excretion of xylem fluid at leaf margins is called guttation, a natural phenomenon. Since the use of neonicotinoid systemic insecticides on seeds reduces the population of bees drastically and causes significant losses in the plant pollination phenomenon, the use of this particular chemical was banned by the European Union [71]. On the contrary, a well-known bio-insecticide, *Bacillus thuringiensis*, which works harmoniously with plants, is responsible for controlling a broad range of diverse insects for pest management in agricultural fields [72]. Some other examples are *B. cereus*, *B. subtilis*, and *B. amyloliquefaciens*, which are also involved in pest control and management [73].

## 6. Perspectives and future of *Bacillus*-based products

**Figure 2.**
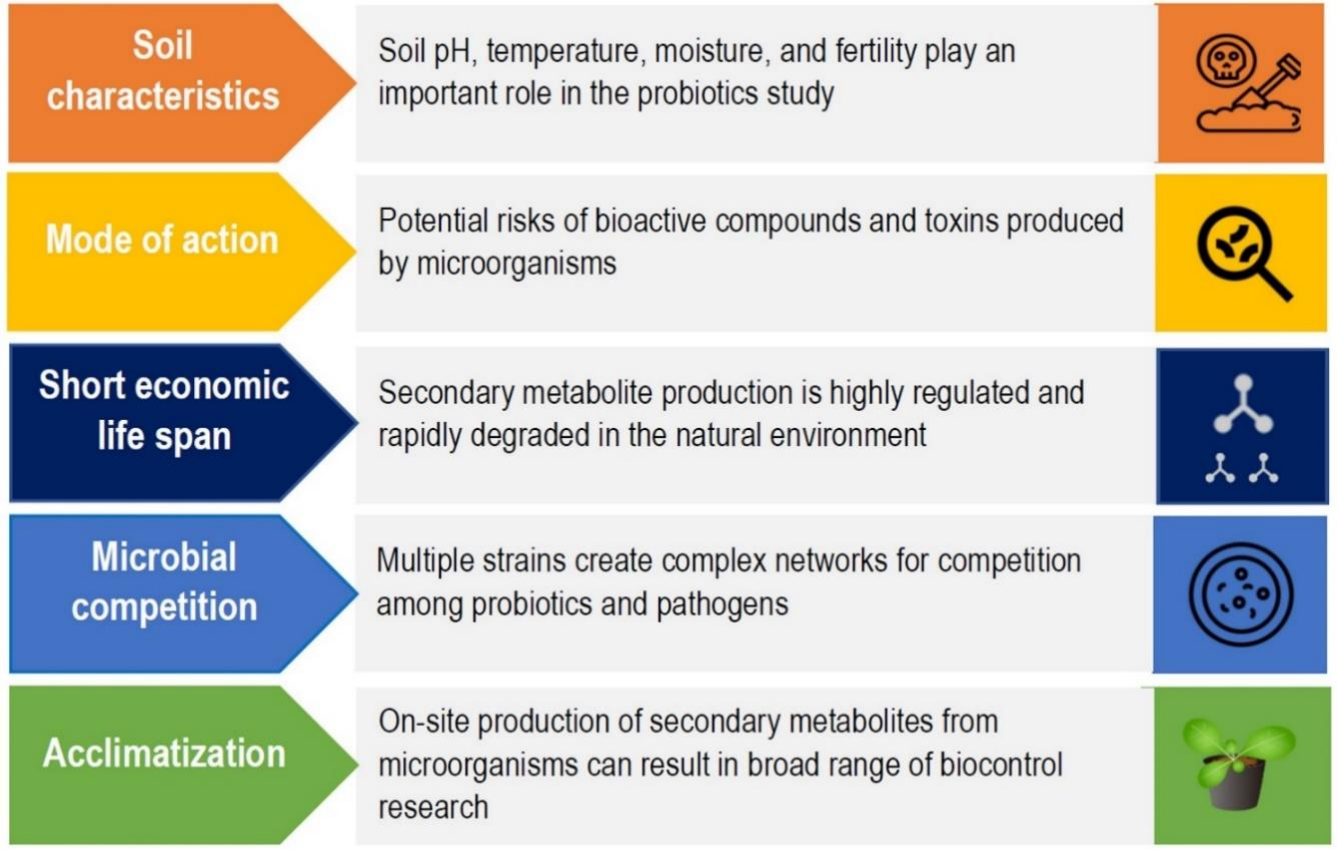
Limitations with probiotic efficiency and stability. Schematic representation of the challenges and limitations associated with the probiotic efficiency and stability of plant probiotics. Relevant factors include soil characteristics, mode of action, short economic life span, microbial competition, and acclimatization.

There is a shred of clear evidence from previous studies that applying probiotics has long-term effects on crop productivity in terms of improving quality and quantity. However, due to their complex nature and lesser popularity, a solid application strategy is needed to promote the usage of probiotic bacteria consortiums in agriculture, especially in developing countries. Studies on the mode of action show antagonistic behavior towards pathogens, but the efficacy is decreased during cascades of physiological events (Figure 2). With the development of novel probiotic-based fertilizers, specific modes of action might be considered. In that case, robust screening of novel *Bacillus* species as probiotic strains with potential antimicrobial metabolites can be used. This screening method may work well with the superior kind of strains and the overall effects of the pathogen and disease. In terms of future research, a few aspects should be given immediate consideration:

- Recommendation of precision probiotic bacteria fertilizer: The precision fertilizer, which is selected based on soil mineralogy and dynamics, is of utmost importance to improve crop yield and cost-effectiveness. Initially, *in vitro* experimental setup should be brought up in the fields by selecting certain districts or counties in a country. A certain number of soil types must then be chosen as samples for mineralogy and dynamics and observed to compare conventional kinds of fertilizers.
- Crop quality consideration: Soil quality plays a vital role in the improvement of the quality of most crops. However, for precise and more accurate crop-responsive probiotic application, a suitable formulation might be used to investigate the dynamicity in the system, minimizing the use of chemical fertilizers and promoting organic agriculture for a sustainable environment. Moreover, public sector research institutions, fertilizer companies, and agriculture-based industries should work together to develop a research and awareness strategy for the promotion of probiotics bacteria in agriculture.
- Multi-omics approach: better screening assays such as multi-omics inclusive of genomics, proteomics, and transcriptomics are required to find the next-generation probiotic strains among all the *Bacillus* species.

Every year, commercial treatments concerning novel bacterial inoculants for different crops are growing. Government clearance for novel inoculants and formulations is not always universal and well-established; therefore, the commercialization process requires more attention. Additionally, the commercialization and registration of new products depend on the country [74], and rules and regulations vary according to the country. Until now, no international agreement on biofertilizer utilization and quality control in agricultural and horticultural industries has been established. According to most of the protocols established by North America, a novel formulation, including *Bacillus* species either using organic or inorganic carriers, must be safe and non-toxic for the environment and human health. Current commercial *Bacillus* sp. products are listed in Table 3 [75]–[77]. However, a few species, such as *B. cereus*, are considered pathogenic to humans and require clearance and registration for large-scale production [78],[79].

In conclusion, this perspective not only provides a synthesis of current knowledge on the utilization of *Bacillus* species in challenging agricultural environments but also serves as a catalyst for future research endeavors. The continued exploration of the intricate interactions between *Bacillus* species and plants holds promise for advancing our understanding of plant probiotics and their pivotal role in shaping the future of agriculture under adverse conditions.

**Table 3.** Commercial biofertilizers brands in the market with bacterial strains.

**Inoculators**	**Product**	**Plants**	**Viability**	**Company**
*B. amyloliquefaciens, Bradyrhizobium japonicum*	Nodulator^®^ N/T Peat	Increase the yield of soybean	24 hours on-seed survivability	BASF Canada Inc.
*Bacillus amyloliquefaciens, Bradyrhizobium japonicum*	Nodulator^®^ PRO 100	Soybean fungicides	24 hours on-seed survivability	BASF Canada Inc.
*Bacillus subtilis, Bacillus amyloliquefaciens*	PrimAgro^®^ C-Tech	Pest control in all types of crops	6 months	Canadian Corporation
*Bacillus subtilis, Bacillus amyloliquefaciens*	BioSoil Enhancers, Inc	Pest control in all types of crops	6 months	Arizona Corporation
*Bacillus amyloliquefaciens, Bacillus licheniformis, Bacillus megaterium, Bacillus pumilus, Bacillus subtilis, Trichoderma*	BioSafe^®^ Systems	Pest control in all types of crops	6–8 months	Arizona Corporation
*Bacillus subtilis*	BioWorks^®^	Pest control in all types of crops	-	Arizona Corporation
*Bacillus amyloliquefaciens, Bacillus licheniformis, Bacillus subtilis*	Blacksmith Bioscience^®^	Pest control in all types of crops	6 months	Arizona Corporation
*Bacillus subtilis*	Concentric Ag^®^	Pest control in all types of crops	6 months	Arizona Corporation
*Bacillus amyloliquefaciens, Bacillus subtilis, Pseudomonas monteilii*	Earth Alive^®^	Pest control in all types of crops	-	Arizona Corporation
*Bacillus subtilis, Bacillus amyloliquefaciens*	Impello Biosciences^®^	Pest control in all types of crops	-	Arizona Corporation
*Bacillus subtilis*	John and Bob's^®^	Pest control in all types of crops	-	Arizona Corporation
*Bacillus subtilis, Bacillus amyloliquefaciens*	Key to Life^®^	Pest control in all types of crops	-	Arizona Corporation
*Bacillus subtilis*	SCD Probiotics^®^	Pest control in all types of crops	-	Arizona Corporation

## Use of AI tools declaration

The authors declare they have not used artificial intelligence (AI) tools in the creation of this article.
